# Distribution and drivers of global mangrove forest change, 1996–2010

**DOI:** 10.1371/journal.pone.0179302

**Published:** 2017-06-08

**Authors:** Nathan Thomas, Richard Lucas, Peter Bunting, Andrew Hardy, Ake Rosenqvist, Marc Simard

**Affiliations:** 1 California Institute of Technology Jet Propulsion Laboratory, Pasadena, California, United States of America; 2 Centre for Ecosystem Science, The University of New South Wales, Sydney, Australia; 3 Department of Geography and Earth Sciences, Aberystwyth University, Aberystwyth, Wales, United Kingdom; 4 Solo Earth Observation (soloEO), Tokyo, Japan; Kerala Forest Research Institute, INDIA

## Abstract

For the period 1996-2010, we provide the first indication of the drivers behind mangrove land cover and land use change across the (pan-)tropics using time-series Japanese Earth Resources Satellite (JERS-1) Synthetic Aperture Radar (SAR) and Advanced Land Observing Satellite (ALOS) Phased Array-type L-band SAR (PALSAR) data. Multi-temporal radar mosaics were manually interpreted for evidence of loss and gain in forest extent and its associated driver. Mangrove loss as a consequence of human activities was observed across their entire range. Between 1996-2010 12% of the 1168 1°x1° radar mosaic tiles examined contained evidence of mangrove loss, as a consequence of anthropogenic degradation, with this increasing to 38% when combined with evidence of anthropogenic activity prior to 1996. The greatest proportion of loss was observed in Southeast Asia, whereby approximately 50% of the tiles in the region contained evidence of mangrove loss, corresponding to 18.4% of the global mangrove forest tiles. Southeast Asia contained the greatest proportion (33.8%) of global mangrove forest. The primary driver of anthropogenic mangrove loss was found to be the conversion of mangrove to aquaculture/agriculture, although substantial advance of mangroves was also evident in many regions.

## Introduction

Mangrove forests are located in the tropics and sub-tropics but extend into temperate regions where they reach their geographical limits [1, 2]. They provide many ecosystem services, such as support for local livelihoods through the provision of fuel, food and construction materials [3]. Mangroves host a wide variety of biodiversity, providing habitats for fauna including aquatic and terrestrial insects, fish, crustacean, mammalian, amphibian, reptilian and avian species [1]. Mangrove forests contain very high levels of above ground biomass (AGB) as well as high quantities of below ground biomass [4], which equates to carbon storage levels [5–7] equivalent to those measured in dense Amazonian rainforests [8]. This makes mangroves amongst the most carbon rich ecosystems in the tropics [9]. The high carbon content of mangroves, coupled with their financial value in terms of the ecosystems services that they support, makes them an important asset for carbon trading initiatives (e.g., REDD+) [10].

Despite their importance for local livelihoods, biodiversity and carbon sequestration, mangrove forests are greatly threatened across their range. Mangrove losses over the period 1980-2005 were estimated to be > 3 million ha^10^ with the rate of degradation over the period 1990-2000 estimated at 1% per year, a rate twice that of terrestrial rainforests [11]. The release of carbon into the atmosphere as a consequence of mangrove degradation has been estimated to be 0.02-0.12 Pg per year, representing 10% of total emissions resulting from deforestation [9]. The primary cause of loss has been conversion to aquaculture practices, the fastest growing animal-food sector in the world [12]. Developing countries have dominated this production, with the Asia-Pacific region accounting for almost 90% of global production [13].

Mapping and monitoring the changing extent of mangroves has proved difficult, with most attempts relying on a combination of remotely sensed data and existing field maps to catalogue mangrove extent for a single point in time. Spalding et al. [14] produced a global map (updated in 2010; [15]) from a range of inventories with varying degrees of quality including georeferenced sketch maps, some of which were dated to the 1960s. Similarly, a global assessment of mangrove forests conducted by the FAO [16, 17] produced an inventory of mangrove extent across the globe but offered no geographical interpretation of their distribution. As such, these inventories offer no insight into the changes in the spatial distribution of mangrove extent. Spatially explicit maps of mangrove extent, consistently at a single point in time, are required to direct and inform the management and conservation of mangrove forests.

The first mangrove map of the globe to utilize remotely sensed data was produced by Giri et al. [18] who applied a combination of automatic and manual classification techniques to over 1000 Landsat scenes, acquired between 1997 and 2000. Although a global map was achieved, the accuracy of the product was not provided, presented a single snapshot in time and was unable to capture important information on the spatio-temporal dynamics of mangrove forests. Changes in mangrove forest extent were mapped for the first time at the global scale by Hamilton and Casey [19] for the period 2000-2012. A combination of existing products were used, including the mangrove map of Giri et al. [18], Global forest Change maps of Hansen et al. [20] and terrestrial eco-region datasets [21]. This intersected the mangrove map with forest change maps to reveal changes that had occurred specifically to mangroves. Although this approach led to the quantification of global mangrove gain and loss, it was dependent on pre-existing datasets, without which it could not be readily updated. Furthermore, although the magnitude of change could be estimated, the driver of change was not determined. This was evaluated for Southeast Asia using a combination of the Global forest change map [20] and reference to Google Earth imagery [22], but has not been achieved at the global scale. This knowledge is essential to reliably inform local management and conservation strategies. No changes in mangrove forest extent have been mapped globally prior to the nominal year 2000. Acquiring such information at the global scale is challenging as optical imagery is often inhibited by cloud cover and in many parts of the world is limited in temporal frequency.

Radar data, such as that acquired by Japanese Earth Resources Satellite (JERS-1) Synthetic Aperture Radar (SAR) and Advanced Land Observing Satellite (ALOS) Phased Array-type L-band SAR (PALSAR), offer a benefit over optical imagery in that they have the ability to gather information about the Earth’s surface regardless of weather and illumination conditions. L-band radar (23.5 cm wavelength) offers a unique opportunity for monitoring the changing extent of mangroves across their range in a systematic manner [23]. In many cases, radar data have been acquired systematically across the globe, particularly in the case of Japanese L-band sensors. Time-series of JERS-1 SAR and ALOS PALSAR data over the global mangrove regions are available for the 1995-1998 and 2007-2010 periods respectively [24, 25]. Additionally, recent missions such as ALOS-2 PALSAR-2 and Sentinel-1 (C-band SAR) ensure that radar datasets will continue to be collected in the future, enabling the development of operational monitoring programmes [26]. When used in combination, these datasets provide an opportunity to detect changes in mangrove extent over time.

This study investigates the distribution of observed drivers of change in mangrove forest extent, over the period 1996-2010 using time-series radar composite imagery. This study uses both spatial and temporal information to achieve this novel understanding of changes in mangrove forest extent at the global scale.

## Materials and method

This study undertook an analysis of 1168 1°x1° radar mosaic composite images combining 1996 JERS-1 and 2007 and 2010 ALOS PALSAR mosaic imagery. The data was compiled and provided by the Japanese Aerospace Exploration Agency (JAXA) [27], across the geographical range of mangrove forests. The composite imagery was visually interpreted to determine the distribution of changes in extent over the period 1996-2010. In addition, these composites were used to identify the drivers of the change in land cover and land use change. Validation of the observed changes and their drivers was undertaken with reference to the dense time-series of Landsat optical imagery and high-resolution imagery via Google Earth. The Google Earth Engine Landsat dense time-series enabled changes to Earth’s surface to be monitored over the time-period of the color composite images, at high resolution using both spatial and temporal information.

**Time-series composites** The data for this work was made available for use to the Global Mangrove Watch (GMW), an international coalition conceived through JAXA’s Kyoto & Carbon (K&C) Initiative to demonstrate the potential of time-series Japanese L-band Synthetic Aperture Radar (SAR) for characterizing, mapping and monitoring mangroves at regional to global scales [10]. Time-series of JERS-1 imagery from 1996 and ALOS PALSAR imagery data from 2007 and 2010 were compiled, in KML format for Google Earth, to generate temporal color composite (1996, 2007 and 2010 in Red, Green and Blue bands) tiles measuring 1°x1° in extent at 25 m pixel resolution. Only the HH polarization channel was used. The total geographical extent of the color composite dataset was defined using the mangrove map of Giri et al., [18]. The 1168 tiles were selected from a larger dataset where poor or missing data rendered some tiles unusable. The missing data was concentrated along the coastal zone of China and accounted for 3.5% of the total number of tiles. This loss of data led to 97.5% of the mangrove area defined by Giri et al., [18] being used in this study. The dataset was divided into regions, namely North and Central America (including the Caribbean), South America, Africa, MEI (Middle East & India), Southeast Asia and Oceania (Australia, New Zealand and Pacific Islands).

**Fig 1:**
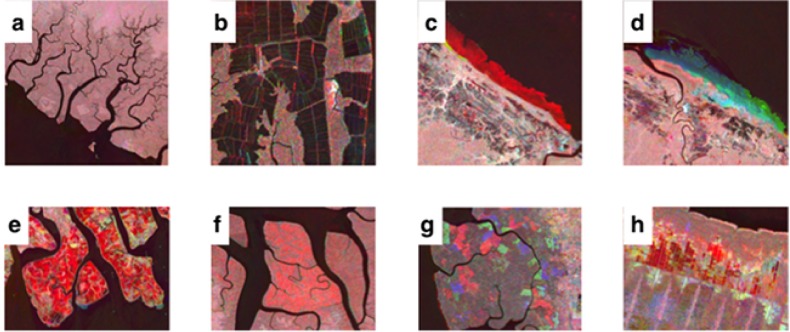
Examples of the categories of change identified within the color composite imagery. Changes in mangrove extent were identified through a combination of their color, their shape and the context of the surrounding environment in the composite imagery. a) intact mangroves in Papua, Indonesia b) prior disturbance (aquaculture) at Guayaquil, Ecuador c) loss of mangrove along the coastline of French Guiana d) colonization of mangrove along the French Guiana coastline e) extensive aquaculture at the Mahakam Delta, East Kalimantan, Indonesia f) mangrove dieback in West Papua, Indonesia g) logging within the managed Matang forest reserve, Perak, Malaysia h) prior and on-going agriculture in Sumatra. Imagery copyright of JAXA.

**Detecting Change** Each region was manually interpreted to identify evidence of changes in mangrove extent to a predefined set of categories. These categories were selected using reference to the literature on the causes of mangrove forest loss [12, 28] in combination with expert knowledge derived from research and publications by members of the GMW, with extensive experience in mangrove environments within North and Central America, South America, Africa, Southeast Asia and Oceania. The categories of change identified were: 1) Intact, 2) Prior Disturbance, 3) Erosion, 4) Deposition/Regrowth, 5) Aquaculture/Agriculture, 6) Dieback 7) Logging. Examples of these processes are shown in Fig 1, which demonstrates that evidence of each process is distinct in the composite imagery due to differences in backscatter over the time series as well contextual information (e.g. smooth texture due to specular reflectance of radar from a water surface and non-natural geometric shape of aquaculture ponds). In addition, regions where large-scale changes in mangrove change had occurred, as a consequence of natural or anthropogenic processes, were identified as ‘hotspots’ which should be prioritized for future monitoring. Computer-vision algorithms capable of this are still in their infancy, therefore human interpretation was used to exploit this contextual information, despite being more time-consuming and potentially subjective. The output is a designation of the whole tile, which can include both multiple categories and multiple occurrences of change. The identification of a single occurrence of change (e.g., an aquaculture pond) was sufficient to label the tile as a change tile. Natural processes of change (e.g. mangrove advance) greater than a single pixel from the coastline was identified as change. No minimum mapping unit for change was considered. Geo-location error between layers was observed in places, yet was recognized by the interpreter as a consistent colored border along the coastline and was subsequently differentiated from a change occurrence.

In L-band SAR imagery the backscatter of the incident energy to the sensor is dependent upon the physical structure of the land cover being imaged. Larger structures increase backscatter so that the large vertical structure of a mangrove results in an increase in backscatter over grass, which has a smaller structure. Intact mangroves appear brighter (medium to high backscatter) in radar imagery than smaller vegetation and open water surfaces which appear dark (low backscatter). Intermediate growth stages have backscatter that increases with vegetation size. As mangrove forests and rainforests are structurally similar, gradual natural changes at the landward margin or within the mangrove forest could not be deciphered due to similarity in backscatter between images.

Each channel of the temporal color composite represents a specific point in time (red: 1996, green: 2007, blue: 2010), therefore the composite imagery provides a means of inferring changes to the mangrove extent through time. A decrease in backscatter from 1996, synonymous with the removal of mangroves, is observed as a distinct red region because of the high backscatter relative to 2007 and 2010. Conversely, distinct blue regions were observed as a consequence of an increase in backscatter by 2010, synonymous with the colonization of a section of coastline by mangroves. Distinct regions of green in the imagery are indicative of the colonization and subsequent loss of mangrove over the 14-year period (1996-2010) with increases and subsequent decreases in backscatter. Cyan (2007, 2010), magenta (1996, 2010) and yellow (1996, 2007) were synonymous with degrees of change within this period. The color is used in combination with the context of the observed change, including its location, shape and size. For example, Fig 1C shows a loss of mangrove through erosion, defined by the deep red color, its location on the seaward margin of a mangrove forest and its irregularly shaped boundary. The detection of changes over very short time-periods was challenging. Subsequently, detecting agriculture was dependent upon the period between the removal of the mangrove and growth of the crop/plantation and when the radar data were acquired. Therefore, some mature crops could not be readily distinguished from a mangrove forest.

**Accuracy Assessment** The accuracy assessment was carried out by comparing changes observed in the radar dataset with 30 m resolution Landsat dense time-series imagery from 1984 through to 2012 hosted by the Google Earth Engine and contemporary high resolution Google Earth imagery where available, following Thomas et al. [29]. The use of optical time-series imagery enabled the spatial and temporal validation of the observed driver. Due to the comparable resolution of Landsat (30 m) with ALOS PALSAR (25 m) imagery as well as multispectral capabilities offering insight into specific land cover types, the Landsat dense time-series can be used to efficiently validate observed changes in the color composite radar images, reinforced by the high resolution of current Google Earth imagery. A sample of 30 change tiles (i.e., at least one observation of change identified as loss/gain within a scene) from each change-class were selected at random, with a total of 159 tiles validated. In instances where less than 30 tiles were identified for a class, the total number of tiles for that class was validated. The observed change in the tile was then compared against visual interpretation of the dense time-series Landsat imagery. The accuracy of mangrove dieback could not be determined because this process was not readily identifiable in the time-series of optical satellite imagery and was omitted from the accuracy assessment. In addition, the spatial resolution of the imagery (25 m) limited the detection of mangroves occupying small localized areas. The validation included the ‘Intact’ class, which exhibited no change.

## Results

**Fig 2:**
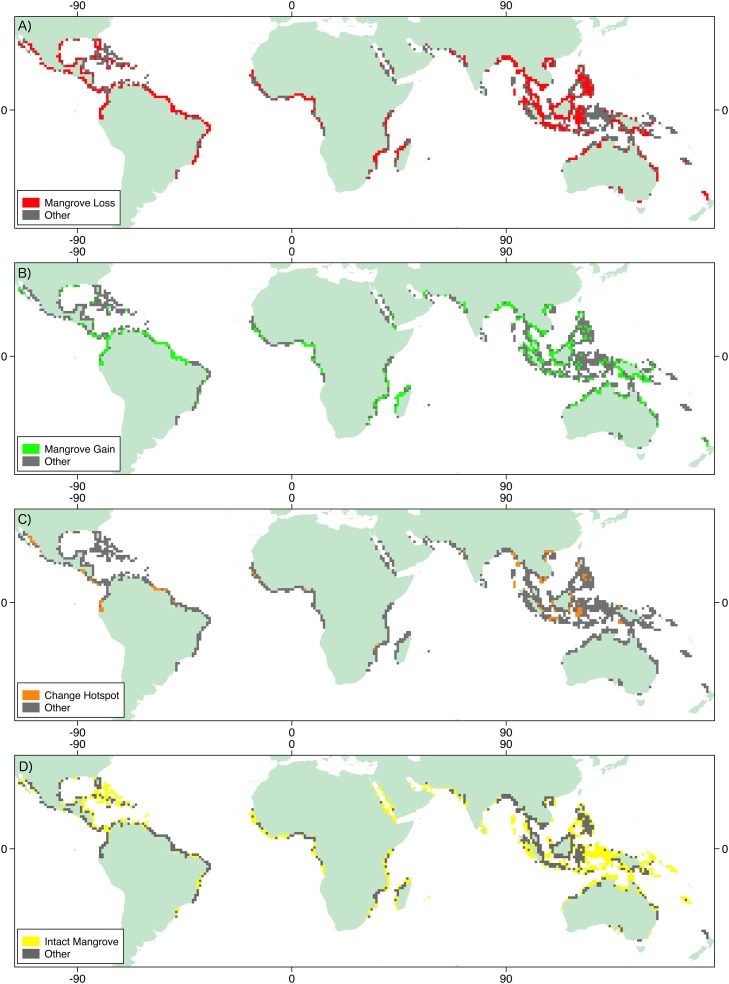
Distribution of different drivers of change in mangrove forest extent across the tropics. A) Advance and regrowth of mangrove extent (1996-2010) B) Degradation from anthropogenic drivers of change including evidence of prior disturbance C) Hotspots where substantial changes in mangrove forest extent were observed (1996-2010) D) Tiles that contained intact mangrove (1996-2010). The total distribution of mangrove tiles is provided in gray.

Changes in mangrove forest extent were observed across their entire range (Fig 2) as a consequence of both natural and anthropogenic drivers of change. No region exhibited wholly intact mangrove extent over the period 1996-2010 with anthropogenic disturbance/removal observed in each of the locations.

**Fig 3:**
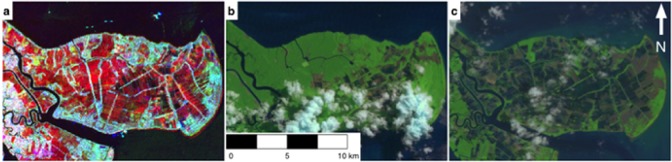
The conversion of mangroves to aquaculture at the Mahakam delta, Kalimantan, Indonesia. Mangrove degradation in the region was observed in the JERS-1/ALOS PALSAR color composite imagery (Red = 1996 JERS-1, Green = 2007 PALSAR, Blue = 2010 PALSAR) and verified using Landsat imagery. a) Color composite SAR image, b) 1996 Landsat 5 TM image, c) 2010 Landsat 5 TM image. The mangrove loss was identified using the distinct color in the radar composite imagery and geometric shape of the change feature. The distinct red color is a consequence of a decrease in radar backscatter in 2007 and 2010 from 1996 due to the replacement of a rough mangrove environment with the smooth surface of an aquaculture pond. Radar imagery copyright of JAXA. Landsat data available from the U.S. Geological Survey.

Losses in mangrove forest extent were observed globally, divided between anthropogenic and natural drivers (Table 1). Regionally, the greatest proportion of tiles where anthropogenic activity was observed was in Southeast Asia (Regional%: 46.7, n = 235, Table 2). Globally, 37.8% of tiles (n = 442) were impacted by such activities, including evidence of activity that occurred prior to 1996. Over the period 1996-2010 the most frequent cause of anthropogenic induced change was the conversion of mangrove to aquaculture/agriculture (11.2% of all tiles, 131 tiles), which was particularly prominent in Southeast Asia (8.3% of the global number of tiles, 97 tiles), followed by logging which was almost exclusive to Southeast Asia. Whilst this identifies the geographical distribution of forest loss, it does not impart the scale on which the practice occurred. Some regions exhibited localized loss (i.e. Panama) whilst aquaculture on a far greater scale was observed in other regions (e.g. Mahakam delta, Kalimantan, Indonesia; Fig 3). These losses were exacerbated by the natural process of erosion which was widespread, but most prominent in Southeast Asia (Regional%: 12.5, n = 59). Erosion was most commonly observed in high-energy environments, such as along exposed coastlines and at the confluence of rivers and at river mouths. Approximately 8% (95 tiles) of the tiles studied were highlighted as ‘hotspots’ of change where intensive changes were identified, as either loss or gain, which should be prioritized for future monitoring. These ‘hotspots’ were located on the West coast of North America, West coast of Central America (Honduras), western coastline of South America (Ecuador), the Bragança peninsula of Brazil, the western coast of Africa (Guinea Bissau, Nigeria), southeast Africa (Mozambique), western India and throughout Southeast Asia (Myanmar, Vietnam, Philippines and Borneo amongst others). The most common cause of change within these tiles was the conversion of mangrove forest to agriculture/aquaculture. Globally, the most frequently observed changes were located in Southeast Asia, whereby 33.8% of the world’s mangroves are located.

**Table 1:** Global mangrove forest change distribution and frequency of mangrove forest change (gain and loss) 1996-2010 by region, as a percentage of the total number of occurrences of change observed globally. NA = North America (including Caribbean), SA = South America, MEI = Middle East and India, SE Asia = Southeast Asia.

Change/Region	NA (%)	SA (%)	Africa (%)	MEI (%)	SE Asia (%)	Oceania (%)	Total (%)
Agri/Aquaculture	1.3	1.2	0.2	0.3	8.3	0	11.2
Erosion	1.8	4.9	3.7	1.5	5.1	3.4	20.3
Regrowth/Deposition	2.1	5.1	5.1	2.1	12.9	5.1	32.4
Logging	0	0	0	0	2.1	0	0.9
Dieback	0.1	0.1	0.3	0	0.9	0.1	1.5
Intact	13.6	5.0	9.2	3.9	29.2	11.0	71.8
Prior Disturbance	7.5	3.9	2.1	1.0	17.3	2.4	34.3
Total Anthropogenic Activity	7.6	4.2	2.3	1.2	20.1	2.4	37.8
Hotspot	1.1	1.3	0.5	0.1	5.1	0	8.1

**Table 2:** Regional mangrove forest change distribution and frequency of mangrove forest change (gain and loss) 1996-2010 as a percentage of the regional occurrences of change, highlighting that lower occurrences in comparison to the global observations still have substantial localized impacts. NA = North America (including Caribbean), SA = South America, MEI = Middle East and India, SE Asia = Southeast Asia.

Change/Region	NA (%)	SA (%)	Africa (%)	MEI (%)	SE Asia (%)	Oceania (%)
Agri/Aquaculture	7.9	11.6	1.3	4.7	20.5	0
Erosion	11.1	47.1	28.3	26.6	12.5	23.8
Regrowth/Deposition	12.6	49.6	39.5	37.5	31.9	35.7
Logging	2.1	0	0	0	0	0
Dieback	0.5	0.8	2.6	0	2.3	0.6
Intact	83.7	47.9	70.4	71.9	72.1	76.2
Prior Disturbance	46.3	38.0	16.4	18.8	42.7	16.7
Total Anthropogenic Activity	46.8	40.5	17.8	21.9	46.7	16.7
Hotspot	6.8	12.4	3.9	1.6	12.7	0

**Fig 4:**
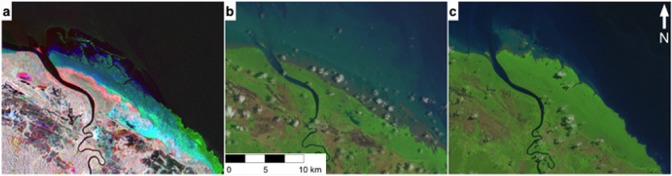
Mangrove advance along the French Guiana coastline. Mangrove advance in the region was observed in the JERS-1/ALOS PALSAR color composite imagery (R = 1996 JERS-1, G = 2007 PALSAR, B = 2010 PALSAR) and verified using Landsat imagery. A) JERS-1/PALSAR color composite image, B) 1997 Landsat 5 TM image, C) 2010 Landsat 5 TM image. The advance was identified due to the coloration of the feature in the radar composite image and its context along the coastline. The distinct blue color is a consequence of enhanced radar backscatter in 2010 due to the rough texture of a mangrove environment over that of the smoother surface of the ocean in 1996 and 2007. Radar imagery copyright of JAXA. Landsat data available from the U.S. Geological Survey.

A total of 379 tiles exhibited colonization/regrowth of which the majority (Regional%: 31.9, n = 151) occurred in Southeast Asia. As with erosion, advance was most commonly observed in high-energy environments as eroded sediment was deposited in sheltered regions along coastlines and within river reaches. An example of this process occurring in French Guiana is shown in Fig 4. The majority of the mangrove was observed to be intact (839 tiles, 71.8%).

Mangrove forest extent is not evenly distributed across the tropics. The total mangrove area defined by Giri et al. [18] used in this study was 134,257 km^2^. The largest proportion of this was located in Southeast Asia (33.8%), followed by Africa (20.9%), with the two regions accounting for over half of the global mangrove forest extent. The distribution of mangrove forest is important in understanding the impact of observed processes of change on the global quantity of mangrove forest. Anthropogenic activity observed in Southeast Asia occurred in tiles that contained 18.4% of the world’s mangrove forest, whereby 12% of the world’s mangrove forest in the region was contained within tiles that were subject to aquaculture/agriculture practices over the period 1996-2010 (Table 3). Oppositely, the mangroves of MEI account for a small proportion of the total mangrove forest extent (6.6%) although practices observed there could have regional importance. Globally, the observed processes of change occurred in tiles with large proportions of the world’s mangrove forest extent. Total anthropogenic loss occurred in tiles containing over 40% of the global mangrove forest, of which 16.1% was due to agriculture/aquaculture over the period 1996-2010. Intact mangrove forest was coincident with 46.5% of the world’s mangrove forest extent.

**Table 3:** Proportional mangrove area contained within change tiles. Area of mangrove forest contained within tiles where change processes were observed. Forest area is provided as a percentage of the global quantity of mangrove forest.

	SA (%)	Oceania (%)	NA (%)	MEI (%)	Africa (%)	SEA (%)	Global %
Anthropogenic loss	4.0	1.7	8.0	4.0	4.6	18.4	40.6
Intact	2.2	6.0	9.8	3.0	9.4	16.3	46.5
Aqua /	1.6	0.0	1.6	0.2	0.8	12.0	16.1
Agriculture Regrowth /	11.6	5.7	3.2	3.6	15.7	20.0	59.7
Deposition Erosion	11.4	4.7	3.4	3.7	10.8	10.4	44.3
Global Area km^2^	18695.8	14236.0	19007.3	8895.9	28105.5	45316.9	
Global Area %	13.9	10.6	14.2	6.6	20.9	33.8	

Using the 1168 tiles, changes in mangrove forest extent were successfully documented across the world with an overall accuracy of 89% (kappa coefficient = 0.87, Table 4) providing a tractable way through which other similar global assessments of land cover change can be conducted.

**Table 4:** Accuracy assessment. Accuracy of identifying a change process within mangrove forest extent 1996-2010.

	Intact	Disturbed Agriculture	Aqua /	Erosion Regrowth	Deposition /	Logging	Total	User’s Accuracy
Intact	28	1	3	4	1	1	38	74
Disturbed	0	29	1	0	0	0	30	100
Aqua / Agriculture	0	0	24	0	2	0	26	92
Erosion	2	0	1	26	0	0	29	87
Deposition/ Regrowth	1	0	0	0	27	0	28	96
Logging	0	0	0	0	0	9	9	100
Total	31	30	29	30	30	10	160	
Producer’s Accuracy		90	97	83	87	90	90	
Total Accuracy	89		kappa	0.87				

## Discussion

This study presents evidence of human-induced land cover and land use change across the extent of the world’s mangroves, supporting previous claims by Dewalt et al. [30], Paez-Osuna [31] and Tong et al. [32] that conversion of mangroves to commercial forms of food and resource production has been widespread. The influence of anthropogenic activities upon mangrove forest extent over the period of observation was substantial, with 11.6% of tiles affected. The majority of loss and degradation, both locally and globally, occurred in Southeast Asia where aquaculture practices were widespread. Globally, 37.8% of tiles were impacted by anthropogenic activities including evidence of activity that occurred prior to 1996, highlighting the impact of historic anthropogenic activity upon mangrove forests. The most common cause of anthropogenic induced change was the conversion of mangrove to aquaculture/agriculture (11.2% of all tiles, 131 tiles). This was exacerbated by the erosion of mangrove forest. This study was not only able to confirm prior observations, but was able to monitor changes in mangrove forest extent across their entire range, identifying the primary drivers of change and their spatial distribution.

Natural processes of erosion and deposition caused mangrove retreat and colonization/regrowth respectively. Erosion and colonization were widespread, occurring in 20.3% and 32.4% of tiles, respectively. Erosion and colonization were commonly observed simultaneously, as along the French Guiana coastline where Fromard et al. [28] measured a change in mangrove extent of 125.4 km^2^ over the period 1951-1999, attributed to both natural processes of erosion and deposition of sediment. A portion of this change was observed in the color composite imagery as illustrated in Fig 4, testament to the use of the method for observing such changes. These processes of change cannot be readily managed as with human induced changes, yet highlight that mangrove forest extent is dynamic across its entire range and is capable of being influenced by external pressures. Upstream urbanization, mining and deforestation affect the influx of sediment into mangrove forests which provide the opportunity for new growth, but which can also be detrimental to some species [33]. Influxes of sediment from urban development and mining can also deliver potentially harmful sediment to existing mangrove forests that can cause estuarine acidification and mangrove death [34].

Although loss and degradation of mangroves between 1996 and 2010 was substantial, this only partially revealed the extent of historic anthropogenic impact upon mangroves. The color composite imagery enabled disturbance that occurred prior to 1996 to be inferred, revealing human activities that replaced entire mangrove forests [35]. This was evident in the imagery by non-natural mangrove boundaries. Examples of this were aquaculture and canals that formed geometric shapes within the mangrove with a black coloration in the composite imagery, as they occurred prior to the earliest image (Fig 1B). Disturbance in excess of 35% of all tiles, reaching regional maximums of 50%, is in keeping with the observed trend of substantial mangrove degradation and loss across their range [36, 37] and supports observations that mangrove forests have undergone historic anthropogenic degradation [12]. This demonstrates the requirement for a global mangrove monitoring system, as changes are occurring on a global scale and over short time periods. It is estimated that over one third of mangrove forest had been lost [12] at the close of the last millennium, yet the distribution of this loss and its drivers have not been spatially documented. Identifying such practices in their early stages is important for mangrove conservation. Whilst knowledge of prior disturbance is important for mangrove rehabilitation, identifying new and rapid degradation of pristine mangrove is of greater importance for mangrove preservation.

The majority of changes, both in overall mangrove extent and as a proportion of the total mangroves found in the region, occurred in Southeast Asia where approximately one-third of mangrove forest area is located. Within this region, industrial shrimp farming has been advocated as a method of obtaining foreign exchange earnings, funding external debt, promoting development, reducing poverty and increasing food security through economic growth in coastal indebted poor countries [38]. This has led to developing nations supporting 90% of global production of farmed seafood consumed worldwide [13], exacerbated by the increasing global demand on marine products and the ability for aquaculture to provide a means of attaining food security, both in terms of national consumption and income generation through exports [39] in developing nations. This is in agreement with Richards and Friess [22] in their assessment of the drivers of mangrove deforestation in Southeast Asia over the period 2000-2012. Aquaculture (shrimp farming) and agriculture (oil palm, rice) were evaluated to be the greatest drivers of mangrove deforestation in the region, with agriculture reported as being more dominant than aquaculture, in contrast to the literature.

It is important that observations of change are evaluated in the context of regional importance. Whilst total anthropogenic disturbance occurred in approximately half of the tiles in Southeast Asia due to its extensive mangrove forest, a similar regional proportion of degradation was observed in North America, due to the historic clearing of mangroves for large urban developments (i.e. Florida; [40]). This highlights that mangrove loss and degradation must be evaluated at the regional scale as the impact of observed changes are proportional to the mangrove area. The frequency of loss and degradation, however, does not impart the areal extent of the degradation as the motive for the removal of mangrove forest can vary from small-scale clearing for localized use to industrial-scale clearing. Furthermore, this highlights contrasting processes behind the drivers of change, whereby some regions may have shifted from mangrove degradation to conservation (e.g., Florida Everglades) in contrast to the current and continued exploitation of mangroves for resources in developing nations (e.g., Southeast Asia).

This study has important implications for mangrove management practices and the ecosystem services that mangrove forests provide. The knowledge on changes in mangrove forest extent and its drivers are critical to understanding the subsequent depletion of mangrove ecosystem services. This is of particular importance in regions of intensive aquaculture whereby the short-term economic incentive of converting a mangrove often outweighs the long-term socioeconomic benefits. Mangroves are capable of generating revenues of US $1,648 y^-1^ × 109 [41, 42] whilst estimates of the annual market value of capture fisheries supported by mangroves is within the range US $750 and US $16,750 ha^1^ [43]. The value of ecosystem services is difficult to quantify, yet large revenues could be attained through the use of mangrove forests within carbon accrediting schemes (REDD+) whilst the protection they provide to coastal communities from tsunamis and storm surges [44] could be considered priceless. This method provides a means of monitoring mangrove change and identifying regions where short-term economic gains are being prioritized over the long-term benefits of mangrove forest conservation, particularly within vulnerable coastal communities. This is particularly prominent in regions of intensive change identified by this study. Approximately 8% (95 tiles) of the total number of tiles were identified as change ‘hotspots’ whereby 7% (82 tiles) were as a consequence of anthropogenic activity. The monitoring of mangroves and their associated ecosystem services should take precedence at these locations. The ability of the method to identify these ‘hotspots’ is critical for informing mangrove management and policy makers to the most threatened reaches of mangrove forest extent. These ‘hotspots’ are the most at risk to the functional loss of the mangrove in terms of the ecosystem services that they provide, which require response from mangrove management initiatives in order to reduce the loss or mitigate against the consequential impacts (i.e., tsunami).

Quantifying the mangrove forest extent that is coincident with process of change is important for understanding the impact that such activities could have on the provision of mangrove forest ecosystem services. Processes that detrimentally impact mangrove extent and health and that affect large proportions of mangrove forest would contribute to the functional loss of the mangrove forest biome, potentially by the end of the century [36]. This information is required by decision and policy makers in order to focus initiatives in regions where large areas of mangrove forest are at risk. The coincident location of 18.4% of the world’s mangroves with observed evidence of anthropogenic activity in Southeast Asia, highlights that a large proportion of mangrove is in an ‘at risk’ region. The preservation of the mangrove environment and its ecosystem services depends upon identifying regions that are at risk from further mangrove degradation, that could continue to negatively impact regions where very large proportions of the global mangrove forest are contained. This study provides a means by which local-scale processes of change can be understood within the context of the global mangrove forest extent. This has led to the important assertion that over half of the mangrove forest in Southeast Asia, where one third of total mangrove forest is located, occurred within a tile with observed anthropogenic activity (aquaculture/agriculture) over the period 1996-2010 alone.

This approach has the potential to be repeated annually with the continued acquisition of JAXA’s ALOS-2 PALSAR-2 and ESA’s freely available Sentinel-1 (C-band) SAR imagery. The accuracy assessment revealed encouraging results (overall accuracy = 89%, kappa coefficient = 0.87), indicating a high degree of correspondence between the L-band radar and Landsat-based assessments of change. This method, therefore, provides a tractable tool for monitoring broad scale land cover changes over the range of mangroves.

## Conclusion

This study provides the first global assessment of the changes and their drivers in mangrove forest extent over the period 1996-2010. Our results reinforce previous claims that mangroves have been historically degraded across their range [12, 37]. The conversion of mangroves to commercial forms of food and resource production has been widespread, observed in 11.6% of tiles. The most common cause of anthropogenic induced change was the conversion of mangrove to aquaculture/agriculture (11.2% of all tiles, 131 tiles) of which the majority occurred in Southeast Asia. Globally, 37.8% of tiles were impacted by anthropogenic activities including evidence of activity that occurred prior to 1996. Natural processes of mangrove loss and advance were frequently observed and widely distributed, occurring in all regions. This study has identified regions of intensive change, in both mangrove gain and loss, that we recommend undergo increased future monitoring. We provide evidence that anthropogenic activities are detrimentally impacting mangrove forests and the ecosystem services that they provide. Emphasis should therefore be placed on quantifying these services in monetary terms, thereby forging an economic counterbalance in the face of heavy pressures from a lucrative aquaculture industry. It is acknowledged that future operational monitoring systems may benefit from automatic satellite based classification and change detection approaches. In particular, the application of SAR data for observing mangrove coastal extent dynamics is recommended.
